# Complete genome sequence of a carlavirus identified in grapevine (*Vitis* sp) in Greece

**DOI:** 10.1007/s00705-023-05795-6

**Published:** 2023-06-01

**Authors:** Konstantina Katsarou, Christos Andronis, Anthony James, Kostas Euthymiou, Nikoleta Kryovrysanaki, Polykseni G. Pappi, Kriton Kalantidis

**Affiliations:** 1 Department of Biology, University of Crete, Vassilika Vouton, Heraklion/Crete, GR-71409 Greece; 2 Institute of Molecular Biology and Biotechnology, Foundation for Research and Technology, Heraklion/Crete, GR-71110 Greece; 3 Laboratory of Plant Virology, Department of Viticulture, Vegetable Crops, Floriculture and Plant Protection, Institute of Olive Tree, Subtropical Crops and Viticulture, Hellenic Agricultural Organization – “DIMITRA”, 71307 Heraklion, Crete, GR Greece

## Abstract

Using high-throughput sequencing, we identified a novel carlavirus sequence in a 28-year-old ‘Kotsifali’ grapevine sample collected in Heraklion (Crete, Greece). Using RT-PCR and 5'/3' RACE together with Sanger sequencing, the complete genome sequence of 8299 nt was confirmed and found to contain five open reading frames (ORFs) but to lack an ORF6, which is present in some members of the genus *Carlavirus*. The novel sequence is most similar to those of two carlaviruses infecting caper, and taking into account the ICTV nomenclature, we propose the name "grapevine carlavirus 1" for this new virus.

**Supplementary Information:**

The online version contains supplementary material available at 10.1007/s00705-023-05795-6.

*Betaflexiviridae* is a large family of viruses that infect a broad range of plants. It comprises 15 different genera, including the genus *Carlavirus*. Carlaviruses are non-enveloped, filamentous viruses that vary in length between 600 and 700 nm and are 12–15 nm in diameter. Virions contain a single molecule of positive-sense, single-stranded RNA, 7.4 to 8.7 kb in length, containing a 5' cap and 3' polyA tail. The genome typically contains six open reading frames (ORFs), with ORF1 encoding a 215–225 kDa protein functioning as the viral replicase. ORF 2, 3, and 4 encode the conserved triple gene block (TGB) proteins, while ORF5 encodes a coat protein of 32–36 kDa, and ORF6 encodes a cysteine-rich protein (11–16 kDa) with RNA binding activity. All carlaviruses are transmitted mechanically, while several are also known to be transmitted by insect vectors [1].

Grapevine (*Vitis* sp.) is host to the largest number of viruses among the cultivated plant species [2]. At least 86 viruses have been identified in grapevine, including 17 different *Betaflexiviridae* members. These include some economically important viruses such as grapevine virus A, grapevine virus B, and grapevine rupestris stem pitting-associated virus. However, none of the known *Betaflexiviridae* members infecting grapevine belong to the genus *Carlavirus*. In this article, we describe the identification and characterization of a novel carlavirus identified in grapevine in Crete.

In 2019 and 2020, ~ 400 grapevine samples were collected from the four prefectures (Chania, Rethymnon, Heraklion, and Lasithi) of the island of Crete, Greece. Selected samples, mostly from grapevines older than 10 years, were pooled and sequenced using high-throughput sequencing (HTS). One of these pools comprised five samples of the local grape cultivars Vilana (two plants), Kotsifali (two plants), and Mandilari (one plant), all grafted onto 140 Ruggeri rootstock, from a single location in the prefecture of Heraklion. The Vilana grapevines were 36 years old, while the Kotsifali plants were 28 years old, and the Mandilari plant was 10 years old.

For sequencing, total RNA was extracted from leaves using a Spectrum^™^ Plant Total RNA Kit (Sigma-Aldrich), including an on-column DNase I (Roche) digestion, and 1 µg of RNA from each sample was pooled for HTS. Library construction was performed using a TruSeq^®^ Stranded Total RNA with Ribo-Zero^™^ Plant Kit (Illumina) following the manufacturer’s protocol, and 44,461,174 paired-end reads of 101 bp were generated. Read quality was assessed using FastQC [3], quality and adapter trimming were performed using fastp [4], and ribosomal RNA sequences were removed using BBDuk [5] with the default settings and the reference file (ribokmers.fa) included in the BBTools package. Host-derived sequences were removed by alignment with the genome sequence of *V. vinifera* (GenBank no. GCA_000003745.2) using BBSplit [5], applying the default settings. The remaining reads were then assembled *de novo* using SPAdes [6] with the -meta option. Assembled contigs larger than 200 nt were subjected to a BLASTn search (task -blastn, e-value 1E-5) using NCBI BLAST + v. 2.9.0 [7] against the Reference Viral Database (RVDB) v20.0 [8]. Taxonomic information about the BLAST hits of interest was added using the taxonomizr package [9].

BLAST analysis showed that 43 contigs had a high degree of similarity to plant virus or viroid sequences, including those of several well-characterized viruses, like grapevine leafroll-associated viruses 1, 4, and 6, grapevine Pinot Gris virus, and grapevine Roditis leaf discoloration-associated virus, as well as the viroids grapevine yellow speckle viroid 1 and hop stunt viroid (Supplementary Table S1). One contig of 8282 nt appeared to represent the nearly complete genome sequence of a novel *Betaflexiviridae* member, with only 70.5% nt sequence identity to the carlavirus caper latent virus (CapLV). To confirm the presence of the novel sequence in the pooled samples and to determine the full-length genome sequence, a series of primers were designed (Supplementary Table S2) and used for RT-PCR. cDNA was generated using 500 ng of total RNA and random primers using M-MuLV reverse transcriptase (Minotech, Greece) according to the manufacturer’s instructions. PCR was then carried out using Taq DNA polymerase (Minotech, Greece). Initially, the primer pair Carla-D-F/Carla-D-R (Supplementary Table S2) was used to determine which sample(s) within the pool contained the target sequence. Sample number 3 from cultivar Kotsifali produced the expected PCR product of 860 nt, while no amplification was observed when the other four samples were tested (Fig. 1A). The amplicon was excised and sequenced by the Sanger method (Genewiz, Germany), and the sequence of the amplified fragment was 99% identical to the HTS contig. Next, PCR and sequencing of the complete viral genome were carried out using the remaining primer sets (Supplementary Table S2). PCR with each primer pair generated an amplicon of the expected size, which was sequenced as described above (Supplementary Fig. S1A). To confirm the sequences of the 5' and 3' ends of the viral genome, 5' and 3' RACE were carried out using a SuperScript™ III One-Step RT-PCR System (Invitrogen, USA) according to the manufacturer’s protocol, and amplified fragments were sequenced.

**Fig. 1:**
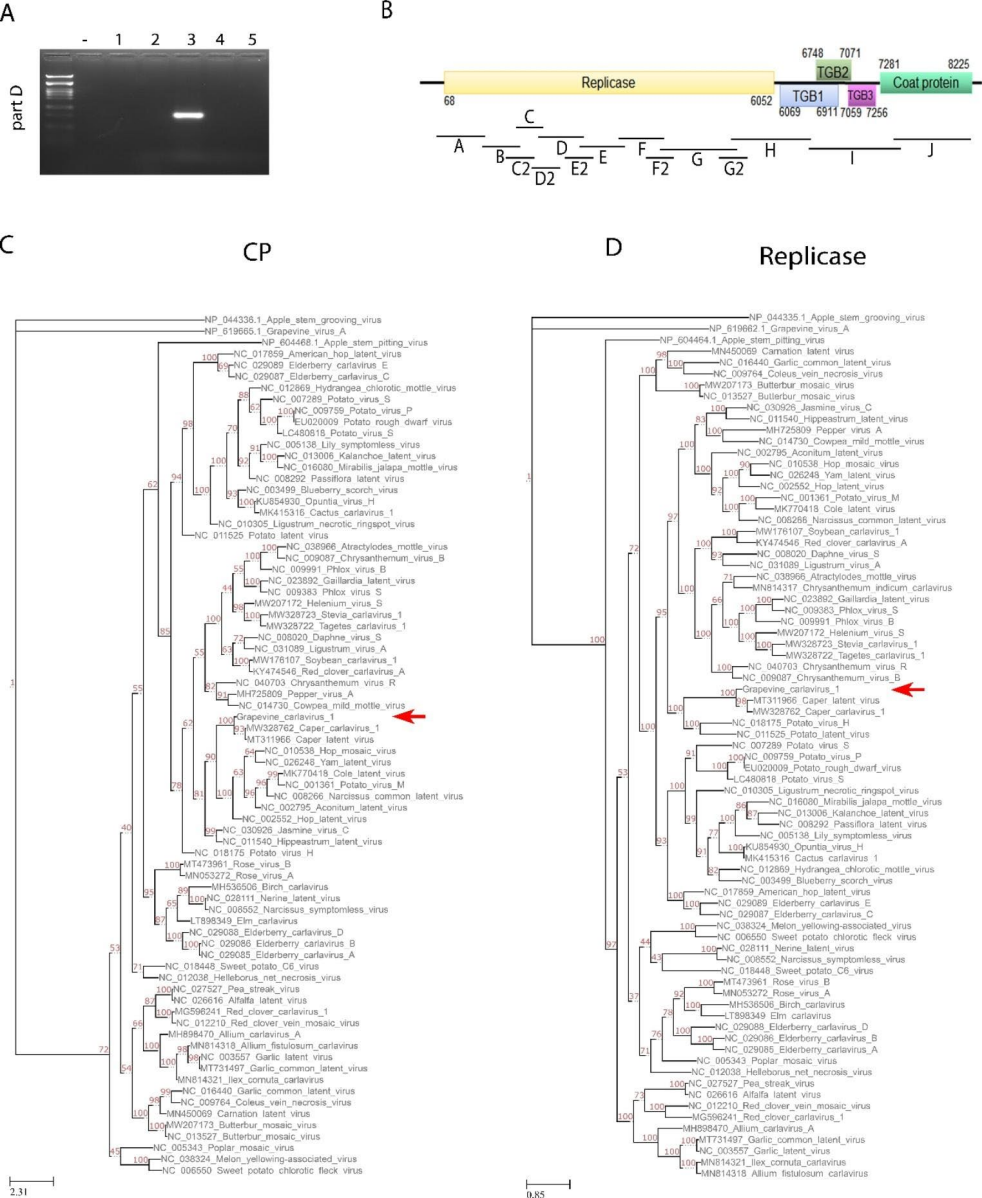
Identification and characterization of a carlavirus from grapevine in Crete. (A) PCR screening of the five pooled grapevine samples to identify the source plant of the novel carlavirus sequence (using primers for part D, see Supplementary Table S2). (B) Schematic representation of the complete genome of grapevine carlavirus 1. Bars correspond to the overlapping PCR fragments that were amplified and sequenced by the Sanger method to verify the HTS results (results in Supplementary Fig. S1). (C and D) Phylogenetic trees based on amino acid sequences of the CP and replicase proteins, using MUSCLE and the maximum-likelihood method with 1000 bootstrap replicates

The complete genome sequence of the novel virus was compiled from the overlapping PCR products and RACE sequences and comprised 8299 nt, excluding the polyA tail (GenBank no. OQ363854). Comparison of the original HTS contig and the Sanger sequence revealed they were almost identical (99.94%). Putative ORFs were identified using ORF Finder [10], with the complete genome predicted to encode five ORFs. ORF1 encodes a polyprotein of 228 kDa containing motifs for methyltransferase, helicase, and RNA-dependent RNA polymerase (RdRp) activity. ORFs 2, 3, and 4 encode the TGB proteins of 31, 12, and 7.2 kDa, respectively, while ORF5 was predicted to encode a CP of 34.8 kDa. No ORF6 was identified based on this analysis (Fig. 1B).

The nt sequence of the complete genome was compared with published sequences, using BLAST, with the grapevine carlavirus sequence having approximately 72% nt sequence identity to soybean carlavirus 1 and jasmine virus C, as well as approximately 71% nt sequence identity to several other carlaviruses, including CapLV (Supplementary Table S3). Based on the current ICTV guidelines for carlaviruses, a member of a new virus species should possess less than 80% amino acid (aa) sequence identity to all known viruses in its polymerase or CP coding gene (https://ictv.global/report_9th/RNApos/Betaflexiviridae), but concerns about these demarcation criteria have been raised recently [11]. Using the replicase aa sequence, the grapevine carlavirus showed 79% identity to caper carlavirus 1 and 78% identity to CapLV, suggesting that it is a novel member of the genus. Interestingly, the CP aa sequence identity was higher than the proposed threshold, with 87.5% aa sequence identity to caper carlavirus 1 and 86.6% identity to CapLV. Finally, the grapevine carlavirus sequence had less than 65% aa sequence identity to other members of the genus (Supplementary Table S3). Phylogenetic analysis was performed using ModelFinder and IQ-Tree [12, 13] following alignment with MUSCLE. The trees were generated using the maximum-likelihood method, using the LG + F + I + G4 model as the best fit for polymerase and the LG + F + G4 model for CP, with 1000 bootstrap replicates [14], and visualized using TreeView [15]. The replicase and CP sequences of the grapevine carlavirus isolate clustered together with the two caper carlaviruses (Fig. 1C and D), confirming that these are the most closely related viruses in the genus.

Based on the host range and replicase and CP aa sequence identity values, the virus reported in this paper is distinct from other known carlaviruses. Accordingly, we have named it "grapevine carlavirus 1". The complete genome has a typical organization for members of the genus *Carlavirus* although it lacks the small ORF6 found in many members of the genus. Interestingly, genome sequence alignments with the two closest caper-infecting viruses identified an insertion of 266 nt in the TGB genes (nt 6939–7205) in grapevine carlavirus 1, suggesting significant genome variation despite the high degree of similarity in the CP coding region. Importantly, no symptoms were observed in the specific vine sample from which the sequence was obtained (Supplementary Fig. S1B). Further work to identify other viruses in this accession will be useful in case symptoms are observed at another time point during the growing season, as well as for testing the mechanical transmissibility of the virus, which is typical of the genus.

## Electronic Supplementary Material

Below is the link to the electronic supplementary material

**Additional file 1: Supplementary Fig. S1** (A) Overlapping PCRs for characterization and sequencing of the complete genome. (B) Photo of the Kotsifali grapevine from which leaf tissue was sampled

**Additional file 2: Supplementary Table S1** List of viruses identified by HTS

**Additional file 3: Supplementary Table S2** Primers used to characterize the novel grapevine carlavirus

**Additional file 4: Supplementary Table S3** BLAST results of the complete nucleotide (nt) or polymerase and coat protein amino acid (aa) sequences of the novel grapevine virus against both assigned and unassigned members of the genus *Carlavirus*
