# Supplementary figures and images for: Complete genome sequence of a carlavirus identified in grapevine (Vitis sp) in Greece
Source: Arch Virol. 2023 Jun 1;168(6):172. doi: 10.1007/s00705-023-05795-6 (PMC10235145; doi:10.1007/s00705-023-05795-6)

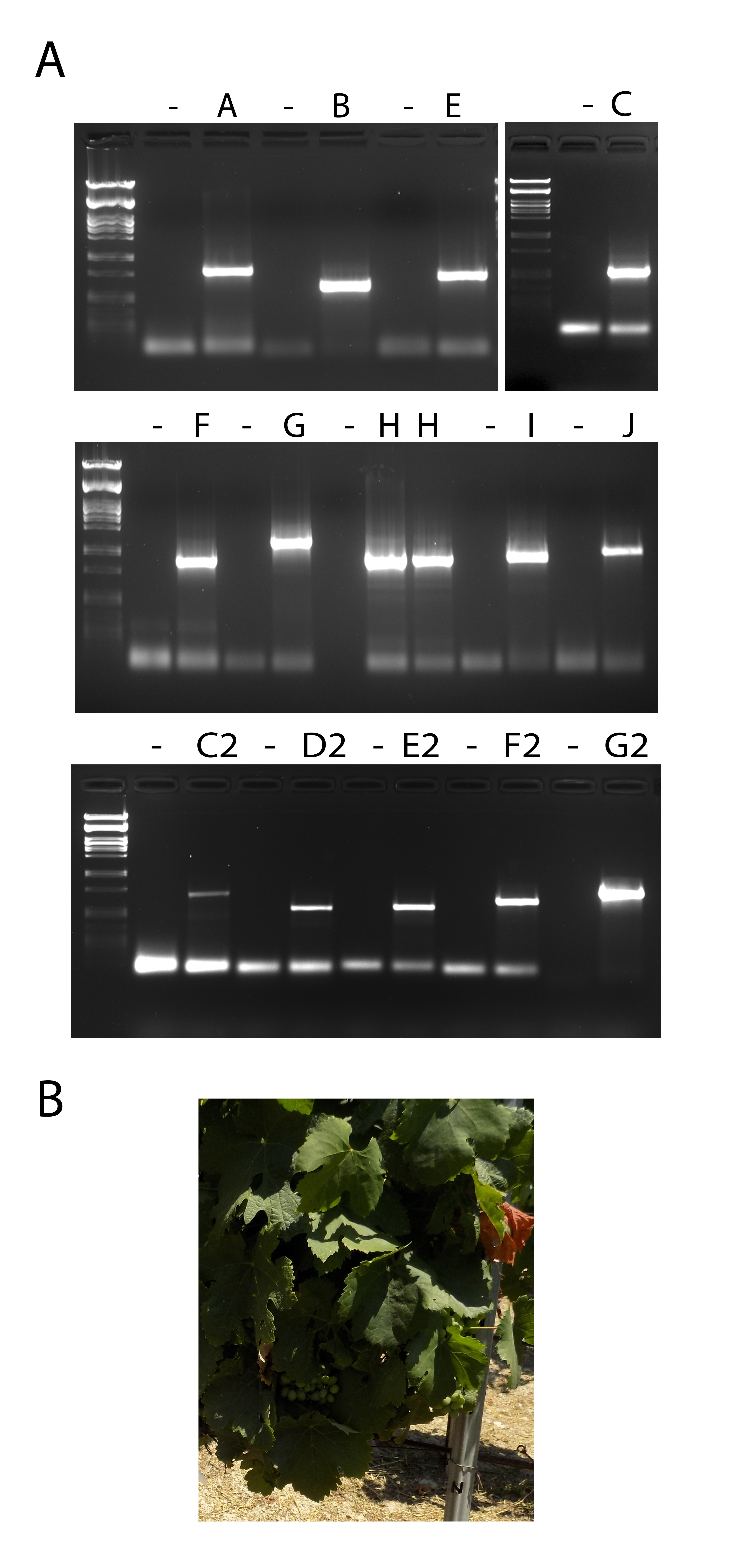

Supplement: Supplementary file 1 — Additional file 1: Supplementary Fig. S1 (A) Overlapping PCRs for characterization and sequencing of the complete genome. (B) Photo of the Kotsifali grapevine from which leaf tissue was sampled [file 705_2023_5795_MOESM1_ESM.tif]
