# Supplementary material for: Complete genome sequence of a carlavirus identified in grapevine (Vitis sp) in Greece
Source: Arch Virol. 2023 Jun 1;168(6):172. doi: 10.1007/s00705-023-05795-6 (PMC10235145; doi:10.1007/s00705-023-05795-6)
Supplement: Supplementary file 3 — Additional file 3: Supplementary Table S2 Primers used to characterize the novel grapevine carlavirus [file 705_2023_5795_MOESM3_ESM.docx]

**Table S2.** Primers used to characterize the novel grapevine carlavirus.

| **Primer name** | **Primer sequence (5**' **- 3')** | **Tm (°C)** | **Amplicon length (bp)** |
| --- | --- | --- | --- |
| **PCR of overlapping fragments** | | | |
| Carla-A-R | AACTTCCATTCCGCAGCTTT | 55 | 860 |
| Carla-A-F | ATACAATATCCTGATAATTCCTAAAGTAAT |  |  |
| Carla-B-F | CAACCGTTGAGTGGGGGTTA | 55 | 627 |
| Carla-B-R | TTGCAGCACGATCTCTGGTT |  |  |
| Carla-C-F | AACCAGAGATCGTGCTGCAA | 55 | 506 |
| Carla-C-R | CCTGGGGTACACCATTGGAC |  |  |
| Carla-C2-F | GCGATTGCAAAATTGGGTCA | 54 | 752 |
| Carla-C2-R | TCGATGTACAGTGTGTCCCT |  |  |
| Carla-D-F | GGGCCACCAACATGGTTAGA | 55 | 860 |
| Carla-D-R | CTGGACCATCGCCCTATCAC |  |  |
| Carla-D2-F | GCAGCTGTACAGGAAGGACA | 54 | 531 |
| Carla-D2-R | CCAAGCTTCATTGCGTGGAC |  |  |
| Carla-E-F | GTGATAGGGCGATGGTCCAG | 55 | 768 |
| Carla-E-R | CTAGCGGAAGGTCTAAGCCG |  |  |
| Carla-E2-F | CTACTACCCAAGCGAAGAGG | 54 | 542 |
| Carla-E2-R | CGTGTGCTCTGATTTGACTG |  |  |
| Carla-F-F | CTGGGCTGAGGATGAAGCAA | 50 | 1020 |
| Carla-F-R | CCAGCTACGCAAATAACGGC |  |  |
| Carla-F2-F | GACCCTTTTCAATGATGCCC | 54 | 640 |
| Carla-F2-R | GCCCATAGCCACTTTTCTTG |  |  |
| Carla-G-F | GCCGTTATTTGCGTAGCTGG | 50 | 1432 |
| Carla-G-R | CCCCTTTCACCCATGCATCT |  |  |
| Carla-G2-F | TGCTGATGTGGCGCAGATTT | 54 | 780 |
| Carla-G2-R | GCACGCTCCCCCATTTTGTA |  |  |
| Carla-H-F | AGATGCATGGGTGAAAGGGG | 50 | 1077 |
| Carla-H-R | CGATCCTGTGGCTCCTGTTT |  |  |
| Carla-I-F | AAACAGGAGCCACAGGATCG | 50 | 1091 |
| Carla-I-R | CCCCTCTCAGTTGGTTGGTC |  |  |
| Carla-J-F | GTCTGTCTTGGCTTTCTGCAT | 50 | 1192 |
| Carla-J-R | TTTAGGCTTTATAGATGCCCACT |  |  |
| **3**' **RACE** | | | |
| 28V | CGATCCCGGGTTTTTTTTTTTTTTTTTV | 58 | 570 |
| Carla3K-UP1 | GGTGTGCCTACTCAGTATGTGC |  |  |
| 28V | CGATCCCGGGTTTTTTTTTTTTTTTTTV | 60 | 370 |
| Carla3K-UP2 | GAACTACATGTTGACACACGATGC |  |  |
| **5**' **RACE** | | | |
| Carla5K | TGCTTCGTATGGACC | 42 |  |
| 5'RaceK | GTAGATGATGGGGGGGGGGG | 60 | 812 |
| Carla5K-DO1 | CCTTCAGTCCGAACTCCATCTGG |  |  |
| 5'RaceK | GTAGATGATGGGGGGGGGGG | 62 | 559 |
| Carla5K-DO2 | GTGTGGCACCCTCAAGTTGC |  |  |
