# Supplementary material for: Complete genome sequence of a carlavirus identified in grapevine (Vitis sp) in Greece
Source: Arch Virol. 2023 Jun 1;168(6):172. doi: 10.1007/s00705-023-05795-6 (PMC10235145; doi:10.1007/s00705-023-05795-6)
Supplement: Supplementary file 4 — Additional file 4: Supplementary Table S3 BLAST results of the complete nucleotide (nt) or polymerase and coat protein amino acid (aa) sequences of the novel grapevine virus against both assigned and unassigned members of the genus Carlavirus [file 705_2023_5795_MOESM4_ESM.docx]

**Table S3. BLAST results of the complete nucleotide (nt) or polymerase and coat protein amino acid (aa) sequences of the novel grapevine virus against both assigned and unassigned members of the genus Carlavirus.**

| **ICTV accepted virus species** | **Abbreviation** | **Species** | **Accession number** | **Pairwise sequence identity (%)** | | |
| --- | --- | --- | --- | --- | --- | --- |
|  |  |  |  | **Complete genome (nt)** | **RdRp (aa)** | **CP**  **(aa)** |
|  | AcLV | Aconitum latent virus | NC_002795 | 69.51 | 51.34 | 62.94 |
|  | AHLV | American hop latent virus | NC_017859 | 67.97 | 51.74 | 52.27 |
|  | BlScV | Blueberry scorch virus | NC_003499 | 68.96 | 51.74 | 50.40 |
|  | CCV-2 | Cactus carlavirus 2 | MK415317 | 69.01 | 51.57 | 51.43 |
|  | CapLV | Caper latent virus | MT311966 | 70.47 | 78.08 | 86.62 |
|  | CLV | Carnation latent virus | MN450069 | 68.80 | 45.83 | 43.66 |
|  | CVB | Chrysanthemum virus B | NC_009087 | 67.41 | 52.82 | 48.08 |
|  | CoLV | Cole latent virus | MK770418 | 69.36 | 51.89 | 64.14 |
|  | CVNV | Coleus vein necrosis virus | NC_009764 | 67.63 | 46.11 | 39.59 |
|  | CPMMV | Cowpea mild mottle virus | NC_014730 | 70.98 | 53.06 | 55.15 |
|  | DVS | Daphne virus S | NC_008020 | 70.18 | 53.55 | 55.69 |
|  | EBCVA | Elderberry symptomless virus | NC_029085 | 67.97 | 51.67 | 43.62 |
|  | GarCLV | Garlic common latent virus | NC_016440 | 68.53 | 48.24 | 41.22 |
|  | HVS | Hellenium virus S | MW207172 | 68.55 | 49.54 | 54.03 |
|  | HNNV | Helleborus net necrosis virus | NC_012038 | 66.87 | 50.13 | 42.12 |
|  | HpLV | Hop latent virus | NC_002552 | 67.85 | 52.44 | 62.44 |
|  | HpMV | Hop mosaic virus | NC_010538 | 66.89 | 50.36 | 59.03 |
|  | KLV | Kalanchoe latent virus | NC_013006 | 67.01 | 50.55 | 47.20 |
|  | LNRSV | Ligustrum necrotic ringspot virus | NC_010305 | 69.64 | 50.32 | 53.87 |
|  | LVA | Ligustrum virus A | NC_031089 | 70.34 | 54.30 | 55.15 |
|  | LSV | Lily symptomless virus | NC_005138 | 67.90 | 47.75 | 48.57 |
|  | MYaV | Melon yellowing-associated virus | NC_038324 | 67.96 | 45.59 | 35.17 |
|  | NCLV | Narcissus common latent virus | NC_008266 | 69.63 | 52.10 | 63.67 |
|  | NSV | Narcissus symptomless virus | NC_008552 | 68.72 | 45.65 | 45.26 |
|  | NeLV | Nerine latent virus | NC_028111 | 68.90 | 45.25 | 45.67 |
|  | PLV | Passiflora latent virus | NC_008292 | 68.38 | 48.63 | 49.38 |
|  | PeSV | Pea streak virus | NC_027527 | 68.29 | 49.20 | 32.31 |
|  | PopMV | Poplar mosaic virus | NC_005343 | 70.48 | 52.19 | 34.94 |
|  | PotLV | Potato latent virus | NC_011525 | 69.14 | 52.20 | 55.51 |
|  | PVH | Potato virus H | NC_018175 | 71.25 | 51.21 | 60.07 |
|  | PVM | Potato virus M | NC_001361 | 68.60 | 51.46 | 64.14 |
|  | PVP | Potato virus P | LC480818 | 70.83 | 50.88 | 51.43 |
|  | PVS | Potato virus S | NC_007289 | 69.39 | 52.17 | 50.20 |
|  | RCVMV | Red clover vein mosaic virus | NC_012210 | 66.18 | 48.07 | 37.44 |
|  | SLV | Shallot latent virus (Garlic latent virus) | NC_003557 | 70.02 | 48.59 | 41.94 |
|  | SPCFV | Sweet potato chlorotic fleck virus | NC_006550 | 67.01 | 46.48 | 38.35 |

Table S2 continued…

| **Unassigned viruses** | ALV | Alfalfa latent virus | NC_026616 | 67.61 | 48.87 | 35.53 |
| --- | --- | --- | --- | --- | --- | --- |
|  |  | Allium carlavirus A | MH898470 | 70.39 | 47.76 | 41.94 |
|  |  | Allium fistulosum carlavirus | MN814318 | 69.68 | 47.16 | 41.52 |
|  | AtrMoV | Atractylodes mottle virus | NC_038966 | 69.55 | 51.08 | 46.82 |
|  | BiCV-1 | Birch carlavirus 1 | MH536506 | 68.66 | 51.93 | 43.67 |
|  | ButMV | Butterbur mosaic virus | NC_013527 | 70.01 | 48.43 | 44.13 |
|  | CCV-1 | Cactus carlavirus 1 | MK415316 | 69.39 | 49.77 | 52.86 |
|  |  | Caper Carlavirus 1 | MW328762 | 71.39 | 79.03 | 87.58 |
|  |  | Chrysanthemum indicum carlavirus | MN814317 | 68.93 | 52.69 |  |
|  | CVR | Chrysanthemum virus R | NC_040703 | 67.29 | 52.46 | 54.09 |
|  | EBCVB | Elderberry carlavirus B | NC_029086 | 70.56 | 51.68 | 43.21 |
|  | EBCVD | Elderberry carlavirus D | NC_029088 | 70.10 | 51.10 | 44.62 |
|  | EBCVE | Elderberry carlavirus E | NC_029089 | 69.21 | 52.35 | 51.47 |
|  | ElmCV | Elm carlavirus | LT898349 | 70.10 | 52.40 | 41.97 |
|  | GalLV | Gaillardia latent virus | NC_023892 | 70.06 | 52.84 | 47.98 |
|  | HiLV | Hippeastrum latent virus | NC_011540 | 69.52 | 52.93 | 62.85 |
|  | HdCMV | Hydrangea chlorotic mottle virus | NC_012869 | 68.39 | 51.77 | 50.00 |
|  |  | Ilex cornuta carlavirus | MN814321 | 68.58 | 47.77 | 42.55 |
|  | JaVC | Jasmine virus C | NC_030926 | 71.95 | 53.54 | 60.65 |
|  | MjMV | Mirabilis jalapa mottle virus | NC_016080 | 67.73 | 50.32 | 47.79 |
|  |  | Opuntia virus H | KU854930 | 68.82 | 49.36 | 51.02 |
|  | PepVA | Pepper virus A | MH725809.1 | 70.26 | 52.21 | 55.77 |
|  | PhlVB | Phlox virus B | NC_009991 | 69.51 | 51.60 | 52.40 |
|  | PhlVS | Phlox virus S | NC_009383 | 71.01 | 52.22 | 46.18 |
|  | PRDV | Potato rough dwarf virus | EU020009 | 67.77 | 46.15 | 52.38 |
|  | RCCVA | Red clover carlavirus A | KY474546 | 70.35 | 54.30 | 55.40 |
|  |  | Red clover carlavirus 1 | MG596241 | 68.03 | 47.90 | 37.44 |
|  | RVA | Rose virus A | MN053272 | 69.41 | 49.36 | 39.66 |
|  | RVB | Rose virus B | MT473961 | 70.57 | 50.27 | 38.84 |
|  | SCV1 | Soybean carlavirus 1 | MW176107 | 72.13 | 48.63 | 54.43 |
|  | StCV1 | Stevia carlavirus 1 | MW328723 | 69.67 | 51.26 | 52.00 |
|  | SPC6V | Sweet potato C6 virus | NC_018448 | 66.90 | 45.49 | 40.80 |
|  |  | Tagetes carlavirus 1 | MW328722 | 68.91 | 52.28 | 49.60 |
|  | YLV | Yam latent virus | NC_026248 | 68.00 | 52.06 | 59.28 |
